# Historicizing psychedelics: counterculture, renaissance, and the neoliberal matrix

**DOI:** 10.3389/fsoc.2023.1114523

**Published:** 2023-09-21

**Authors:** Mateo Sanchez Petrement

**Affiliations:** Amsterdam School for Cultural Analysis, Faculty of Humanities, University of Amsterdam, Amsterdam, Netherlands

**Keywords:** psychedelics, Mark Fisher, collective set and setting, neoliberalism, personal is political, neuroscience, counterculture, individualism

## Abstract

In this essay, I would like to suggest that the historical transition of psychedelics from an association with *counter*culture to becoming part of the *mains*tream is related to the rise of what late cultural theorist Mark Fisher termed “capitalist realism”—the notion that there is no alternative form of social organization and, as such, capitalism simply *is* reality. For Fisher, the economic and political project of neoliberalism was the main agent behind this re-instauration of capitalist hegemony after its de-stabilization by the convergence of several radical forces at the end of the 1960s and early 70s, of which psychedelic “consciousness-expansion” was one. Thus, historicizing psychedelics within the shifts in political economy and culture associated with the “collective set and setting” of neoliberalism can serve both to understand the current shape and operations of the psychedelic “renaissance” as well as help us retrieve these substance's lost political potential. Concretely, this essay argues that such potential was not inherent to psychedelics but embedded in the political economy of the New Deal order, which supported both the formation of discourses, demands, and hopes based on “the social” and, relatedly, the idea that “the personal is political.” As neoliberalism displaced this object of reference in favor of individualism, the personal was de-linked from the political and the dreams—and the threats—of psychedelic utopianism were successfully defused and forgotten. In the process, concretely, the anti-work and collective dimensions of the psychedelic counterculture have been all but lost as psychedelics have returned to enhance or treat individual brains—while leaving capitalist society unchallenged. In light of our ecological and social predicaments, the famous context-dependence of psychedelics can be a powerful reminder that, contra individualism, the social and political traverse the personal—and thus that to change the self in line with the psychedelic values of love and connection ultimately requires changing the world.

## Introduction

In this essay, I would like to suggest that the historical transition of psychedelics from an association with *counter*culture to becoming part of the *mains*tream is related to the rise of what late cultural theorist Fisher (2009) termed “capitalist realism”—the notion that there is no alternative form of social organization and, as such, capitalism simply *is* reality. For Fisher, the main agent behind this closure of our social imagination has been the economic and political project of neoliberalism which, as he argued in his unfinished book introduction titled *Acid Communism* (2018, p. 1141), “is best understood as a project aimed at destroying —to the point of making them unthinkable —the experiments in democratic socialism and libertarian communism that were efflorescing at the end of the Sixties and the beginning of the Seventies.” As the title suggests with its reference to LSD (acid), Fisher understood psychedelic “consciousness-expansion” as part of the subversive forces that the neoliberal “counter-revolution” had to destroy, capture and bury in order to (re)install capitalist hegemony. From this point of view, “neoliberalism” is more than an economic theory or policy platform emerging from the thinkers convened by the famous “Mont Pelerin Society”—it also stands, more generally, for various socioeconomic developments that have shaped culture, knowledge, and subjectivity according to individualist norms (Gilbert, 2013, 2014).

While psychedelic drugs and their uses were hardly Fisher's primary concern, I consider them as useful objects through which to analyse these historical shifts and as gateways to its forgotten potentials. From this point of view, the fact that disavowing its countercultural legacy and anything reminiscing a “hippie's standpoint” (Sessa, 2014, p. 61) has been a condition of possibility for the “psychedelic renaissance” (Langlitz, 2013; Pollan, 2018) acquires a whole new significance, for the latter's neuroscientific claims to political neutrality hide its ideological alignment with neoliberal realism. Thus, situating the renaissance within the broader “collective set and setting” (Hartogsohn, 2020), or “matrix” (Eisner, 1997), of neoliberal capitalism can give us a new understanding of the deflation of our psychedelic horizon from the countercultural hope that these substances could radically transform capitalist society to the more tempered, expedient, and de-politicized concern with treating or enhancing individuals within it.

Indeed, what I want to track by means of psychedelics is the larger story of the displacement of “the social” as an object of reference and government (Rose, 1996) in favor of the individual. In the United States (the focus of most of the following analysis), this displacement is underpinned by the transition between what American historian Gerstle terms the “political orders” of the New Deal and neoliberalism—the first defined by a conception of capitalism as disastrous if left to its own devices and therefore in need of state regulation in favor of the social or public interest, and the second by idea that market forces needed to be liberated from state controls that were hampering growth, innovation and individual freedom (2022, p. 2). As we will see, the counterculture—and by extension, psychedelia—occupies an ambiguous place in this passage from capitalist crisis to capitalist hegemony. As the dominant narrative has it, its valorisation of individual “authenticity” in many ways broke with the collectivist politics of the New Deal and opened the way to their demise. Nonetheless, the counterculture was also viciously attacked for its own real and perceived association with this and other forms of collective politics in the 1960s—suggesting that the reduction of the counterculture and its aftermaths to an individualist essence obscures both how its multiple potentials were influenced, directed, or blocked by its surrounding context and the full extent of the challenge it posed to capitalist norms. Indeed, inspired by his colleague and friend Gilbert [who has written a crucial piece on “psychedelic socialism” (2017)], Fisher sought to unearth those radical potentials from the simplistic view that “the 60s led to neoliberalism”— arguing that this narrative itself is a symptom of capitalist realism (“there was never really any alternative”). From this point of view, historicizing psychedelics and psychedelic experiences within these shifts in political economy can help us understand not only the broader conditions under which their individualist streaks have become dominant but also those under which their collective or social dimensions could—and maybe can—become activated.

Thus, admittedly, my interest in psychedelics is motivated by a broader interest in social change within which the former serve as both powerful tools and entry points to many debates, theories, histories, and possibilities. Analytically, this motivation has the added value of addressing a gap in the psychedelic literature, which is the lack of concern with the historical embedding of psychedelic cultures within political economy. While Langlitz (2013) and Hartogsohn (2020), for example, have greatly contributed to our understanding of the many uses, trajectories, and transformations of psychedelic culture(s) from the 60s to today, they have not been interested in how shifts related to the neoliberal “matrix” may have affected their shape. Similarly, in the rare cases neoliberalism or capitalism do get mentioned, they appear as either a set of values (such as competitive individualism) with little or no historical grounding (e.g., Plesa and Petranker, 2022) or as relating most directly to concerns over the for-profit “corporadelic” takeover (venture capitalists, patents, rich donors, etc) (e.g., Haustfeld, 2020), or both (e.g., Davis, 2018). One notable exception is the work of Gearin and Devenot (2021) and Devenot et al. (2022), who in much more detailed analyses have greatly contributed to a critical perspective on how current scientific and media discourses on psychedelics reflect neoliberal norms (arguments to which I hope to add here). In all, delving into the history of psychedelics in their relation to the neoliberal matrix can brings these perspectives together, give us a deeper sense of the origins of the developments they discuss, and perhaps help us challenge them and uncover other alternatives.

To this end, I will start by situating psychedelic utopianism within the New Deal order to explain the conditions under which it was possible in the first place and the threat it posed to capitalism—especially in its rejection of the work ethic and its association to other social movements of the time. Then, I will turn to the neoconservative and neoliberal reaction to the New Deal, and the place of psychedelics within it—a symbol for the link between laziness and collectivism, both of which had to be done away with to produce the conditions of individual entrepreneurialism. Finally, I will briefly show how the turn from the social to the individual that characterizes this shift is reflected in the psychedelic renaissance as its dominant neuroscientific (and psychological) discourse contributes to what Fisher termed the “privatization of stress” (2018, p. 684)—the process through which individuals are made responsible for affective distress triggered by external causes often beyond their direct control. The overarching promise of psychedelics is that in their insistence on the contextual nature of experience they challenge the individualizing logic at the core not only of such discourses on mental health but of neoliberalism, or even capitalist modernity, in general (Escobar, 2018). For lack of space, my overview of all these processes cannot be comprehensive, and it is my hope that it is read as a line of research in its early stages. Readers familiar with the multidimensional history of neoliberalism, the “long sixties,” and the demise of social democracy might not find much new in this analysis, but I hope that bringing it to bear on contemporary discussions about the politics of psychedelics can open new questions for further research. As I will mention in the conclusion, many of the questions opened up by psychedelics across this history are of great relevance to our contemporary ecological and social predicament.

## The new deal order

In order to make sense of the counterculture and other social movements emerging in the 60s, it is necessary to understand how they emerged from and in response to the strengths and weaknesses of the political economy of the New Deal. Concretely, it is important to understand how the possibility of governing in the name of “society”—paradoxically as both a counterpoint to the excesses of capitalism and to “save” it at its worst moment—was not the result of “impersonal economic forces” but of class struggle waged by organized labor (quite literally, of the strength of “socialist” or socialist-leaning movements at the time) (Gerstle, 2022, p. 25). Responding to the demands and mobilization of workers in the wake of the tremendous social upheaval and deprivation of the Great Depression in the 1930s, the Roosevelt administration turned to a keynesian-type economy that swooped in to pay for extended welfare provisions, public employment and infrastructure (funded through marginal tax rates inconceivable in our times −75 to 90% on the highest incomes), and institute a collective bargaining system that institutionalized worker's newfound power. This resulted in a certain “class compromise” through which more of the profits of business were distributed to the latter and it also served to boost domestic consumption, quell social unrest and bypass more radical demands (such as worker ownership and democracy). As Gerstle also highlights, this compromise was bolstered (if also limited) by the presence of a mighty competitor on the world stage—Communism—and the need, especially after WWII and the beginning of the Cold War, to prove that capitalism could deliver better lives to its citizens. Altogether, and all too schematically, this situation was largely responsible for the “great compression” of inequality that made the post-war period one of “affluence”—a term that often, including in histories of the 60s counterculture, serves to gloss over these prior political struggles and their gains. Of course, even as this prosperity spread across a greater swath of the population, it was far from evenly distributed, and this was reflected in a rather rigid and exclusionary vision of society.

Perhaps the social rigidity of “straight society” that countercultural movements would come to criticize and oppose is best exemplified by the New Deal's establishment of the “white male breadwinner model” (Jaffe, 2021). This model was, first of all, *white*, as Roosevelt compromised with the Southern Dixiecrats by excluding agricultural and domestic workers (mostly POC) from the collective bargaining system (McAlevey, 2020), Jim Crow segregation was left mostly unchallenged, and many government programs still disproportionally aided white people and excluded black Americans (Katznelson, 2005). It was, secondly, *male*, since it was linked to an idea of male citizenship and participation tied to work, which was defined as what men did for a wage outside of the domestic (female) sphere, and thus, finally, it assumed and helped constitute the nuclear family as we have come to imagine it—where men assumed the role of *breadwinner* (the push for higher compensation often resting on the logic of the “family wage”), and women that of caring mother and wife. It was during this time that the vision of the (white, suburban) nuclear family, while far from universal in fact or even accessibility, established itself fully as the ideal of the American Dream (and as another sign of the superiority over communism). Designed as a form of social “containment,” the family was meant as a safe haven and private bulwark against the uncertainties and anxieties of the external world—communism, nuclear war, alienating corporate and industrial work—but was no panacea, as it also came with heavily entrenched and restrictive gender roles and a sense of rootlessness as many moved away from their old kinship networks to become part of a middle class grounded in homeownership, consumerism, child-rearing and social conformity to their new peers (Tyler May, 1989).

In sum, even as it reduced inequality and extended full civil participation to more working men, the social world projected by the New Deal was a rather rigid and normative one marked by racial segregation, strict gender roles, alienating work outside the home, consumerist conformism, and anti-communist paranoia–certainly not a version of the social we should simply return to [it was also subtended by colonial relations (Bhambra, 2021)]. For our purpose, it suffices to note that we must understand the psychedelic utopianism of the 60s' counterculture as born from the intimate link between the relatively emboldening economy and rather suffocating culture described above, for it produced a maddening dissonance in which the possibilities of a world of plenty were being irrationally misused by a conformist and unjust social order. Instead of spending it on “a world which could be free” (Fisher, 2018, p. 1141), this order squandered its abundance on consumerist, military, and repressive ends. This was a madness youth would grow up in, learn to perceive and expose as such, and try to turn on its head by breaking the firm social and psychological compartmentalization it trapped them in.

## Counterculture and psychedelic utopianism

Putting aside for a minute that “the counterculture” was never a homogenous or unified social movement (Braunstein and Doyle, 2002), it is worth picking up the traditional view popularized by Roszak (1969) that it embodied a widespread critique of “technocratic society” to which the use of LSD certainly contributed. From this point of view, the story goes, the counterculture rebelled not only against the postwar order but against the scientific worldview of modernity itself, understanding it, as Marcuse wrote in 1964, as having “projected and promoted a universe in which the domination of nature has remained linked to the domination of man” (Marcuse, 2002, p. 170). Thus, science had not only rationalized, “disenchanted” and instrumentalized the natural world in the name of efficiency and material accumulation but also submitted people to the same principle. Although multiple realities informed this anti-science stance (most notably the questionable “progress” of the nuclear bomb; see Agar, 2008), perhaps none was as consequential for the counterculture as the de-humanization that seemed to be at the core of the world of work, where people were treated as mere numbers and cogs in the gray, hierarchical, regimented and oppressive machinery of industrial, corporate, and military capitalism. Opposing itself to the dreary repetitiveness and standardization of this “robot society” (as Timothy Leary would call it) in which everyone was destined to become part of an undifferentiated consumerist “mass” competing in a meaningless and alienating “rats race” for image, status, and external validation, the counterculture sought to develop alternative sources of personal satisfaction and social validation. Starting from a critique of the scientific west and its materialism, it is little wonder many sought answers by turning to—frequently eastern—spirituality. Praised for re-enchanting the world and for being conceiving of the self more holistically, spirituality seemed simultaneously a means to reconnect to the wider cosmos. Other people, and to one's inner self by tapping into and unleashing one's “human” capacities for love, play, pleasure and creativity. It is here that we begin to encounter certain tensions between individualism and collectivism that would be reflected in the use of LSD as many turned to it to find a new form of sanity diametrically opposed to that of mainstream society.

At one level, LSD was conceived as a tool to “de-condition” the “cultural self” and find, underneath it, an “authentic” self purportedly free from social conditioning. Thus, the mystical experiences induced by psychedelics withdrew the self from the material world of consumerism and external validation to the internal realm of pure consciousness, granting, through direct contact with the divine, authority to a newfound sense of intrinsic self-worth. Far from irredeemably solipsistic, however, this mysticism also seemed to confirm a deep sense of “oneness” between all people, and with it the notion that beyond and beneath specific cultural and social differences and barriers, all could live in perfect love and harmony. From this point of view, the strategy for social transformation was for everybody to simply free their consciousness of unhealthy social norms and patterns (through drugs or otherwise), and positive—even revolutionary—change would follow. Even at the time, many “politicos” criticized this view for ignoring the need for struggle to ensure and advance the material conditions for such freedom (Lee and Shlain, 1985, p. 109), and over time, many have rightly criticized this stance for ignoring the “specificities of race, class, and gender” that informed it and that it thereby reproduced (Shortall, 2014, p. 189)—after all, most hippies were white, male, and middle class. Thus, received wisdom has it that for all their big and good intentions, hippies in fact ushered in a new era of therapeutic narcissism concerned only with liberating and improving the self and therefore paved the way for neoliberal culture (Lasch, 2018; Ingram, 2020).

While this much is true, and cannot be stressed enough, it is also the case that this narrative overlooks the threat the counterculture was seen to pose at the time, the connections it enabled and often had with other social groups and movements, and how, precisely, its more radical potentials were only fully defused through the installation of a new economic terrain (a process, incidentally, to which other movements succumbed as well; for the case of feminism, see Fraser, 2013). At the time, however, the blurring of boundaries characteristic of psychedelic experience can be said to have contributed to some hippies' reach across the social barriers of the post-war order.

At another level, then, the break with socially imposed identity, if achieved by going *deeper into* the mind, also enabled one to *get out through it* by “blowing it up” and grasping at social rather than individual potentials. For one, the use of LSD posed, as Hartogsohn (2020, p. 224–231) has brilliantly documented, a double challenge to the capitalist (or “protestant”) work ethic—both by producing a lack of motivation for sanctioned modes of work and discipline and, correlatively, on the side of consumption, by providing easy pleasures and gains to the (therefore) “undeserving.” This challenge—and its connection with the “inner self”—was made quite explicit by some, such as countercultural activist Jerry Rubin, who claimed that “drug use signifies the total end of the Protestant ethic: screw work, we want to know ourselves. But of course the goal is to free oneself from American society's sick notion of work, success, reward, and status and to find oneself through one's own discipline, hard work, and introspection” (Quoted in Langlitz, 2013, p. 35). While this rejection of the market economy might have only been accessible to relatively affluent youth who enjoyed enough security to “drop out” of it, the very possibility that many of “the sons and daughters of the nation's elite” (Hartogsohn, 2020, p. 165) would reject work, consumption, and other privileges (however temporarily or partially) seemed both unprecedented and a not inconsiderable jab to the legitimacy and reproduction of the social order—for these kids became “class renegades” in the process (Fisher, 2018, p. 1156). Furthermore, as Farber (2013) has argued (and as Rubin's quote suggests), far from a solipsistic politics of consciousness oblivious to material context, many sought to build lives “not on stoned indifference but on active social engagement and community-oriented hard work” (p. 3) that would create new environments, public spaces (Silos, 2003), “right livelihoods,” and alternative social “games” in line with their values (notably by moving “back-to-the-land” and setting up farms and communes [Melville, 1972]).

Perhaps most importantly, as Marcuse (1969) was quick to suggest (in a combination of psychoanalysis and social theory characteristic of the time), by dissolving their ego's and dis-identifying with the system of domination and authority that advantaged them (to the point of suspending upwards aspirations for the often real risk of downwards social mobility), many a youth's “refusal” of straight society also opened the possibility of forming collective alliances, establishing solidarity, and sustaining an egalitarian spirit beyond narrowly defined social categories (significantly perhaps, longhaired hippies were often derogatorially associated with homeless vagrants, as well as with women, people of color, and communists). At closer inspection, then, the security—or “affluence”—many in the counterculture enjoyed not only permitted individual exploration but also supported a strong collectivist and communitarian spirit for which they were not only drawn to mysticism and eastern religions, but also to different forms of activism. It was in the uneasy “convergence” between such new forces of social change that Fisher saw a powerful challenge to the status quo and as a sign of a collective “postcapitalist desire” (Fisher, 2012) before and beyond capitalist realism (2018, p. 1150).

While I do not wish to paint a rosy and certainly untrue picture of harmony amongst the many social movements emerging at the time, it is nonetheless true that “the counterculture,” psychedelic consciousness-expansion, and countercultural modes of expression and dissidence frequently intersected with and informed various forms of “New Left” politics—Student activism, the Women's Movement, Civil Rights, Black Power, Native American “Red Power” (Smith, 2012), and a slowly nascent ecological movement, which perhaps came together most clearly in anti-war demonstrations (Lee and Shlain, 1985; Bach, 2013). Again, while the main divide concerned the necessity of or turn away from more or less traditional forms of agonistic struggle, it can be useful to think of all these movements as “countercultural” in a broad, purely descriptive sense, as they certainly opposed the mainstream society of their time, tried to produce a new culture that could change its values and views (on race, gender, democracy, nature, mental illness, and so on), and sought to prefigure the transformations they desired to bring about through their lifestyles. From this point of view, psychedelics were just another tool for “consciousness-raising”—a task even some drug dealers understood themselves to be undertaking (Lee and Shlain, 1985, p. 116).

In the context of the women's movement, who popularized the term, consciousness-raising referred to the novel practice of coming together in small groups to openly discuss and pool ostensibly “private” experiences to find their common—political—causes, serving the “super-therapeutic” function of overcoming personal alienation and becoming collectively empowered to challenge them (Gilbert, 2017). Evidencing its links to psychedelia, Redstockings member Kathie Sarachild's “program for consciousness-raising”—prepared for the First National Women's Liberation Conference in Chicago, 1968—even referred to the practice as “ongoing consciousness-expansion” (Sarachild, 1968; see also Michals, 2002). In a broader sense, however, consciousness-raising was also part of what the Black Panther Party understood its community programs to be for (The Dr. Hey P. Newton Foundation, 2008), and can also be applied to the “free” services, media stunts, and guerrilla theater tactics of the Diggers and the Yippies, or sit-ins, teach-ins, and voter registration initiatives of the student movement. In all, many of these forms of activism shaped a powerful common sense that the “the personal is political” (another notion pre-existing, but popularized at the time by feminists). By addressing each person as an agent and site of political struggle, such a conception of the self amounted to an elementary form of democratization which called upon each individual to consider and make decisions of broader social significance. In this scenario, as Fisher suggested, psychedelics are quite unique for having democratized both neurology and metaphysics—at once linking the nature of the self to that of its surrounding reality, providing a first-hand “altered” experience of their transmutability, and opening them to questioning and intervention. As Carl Oglesby, former president of Students for a Democratic Society (SDS) described it, even if the “actual content” of the LSD experience was not inherently linked to revolution, “nothing could stand for that overall sense of going through profound changes so well as the immediate, powerful and explicit transformation that you went through when you dropped acid,” and as such, “the experience shared the structural characteristics of political rebellion” (Quoted in Lee and Shlain, 1985, p. 108). In all, while disagreements certainly existed about whether changing consciousness was sufficient to change the world, that it was necessary—and desirable—to do so was a rather consensual matter.

Grouped this way, the term counter-*culture* also helps us makes sense of what these movements did *not* oppose—the great gains in equality achieved by the labor movement and a comparatively social-democratic government. In fact, if anything, most of them took these gains for granted and sought to pursue the cause of equality and the critique of capitalism further both theoretically—producing novel understandings of how capitalism operated (domestic and international racial imperialism, women's reproductive labor in the family, normalizing institutions such as the university or psychiatry, exploitation of nature…)—and practically—calling for additional material redistribution and new forms of “participatory democracy” (a term popularized by SDS) that would increase the groups involved in collective decision making and extend the spheres in which it took place (to the workplace, for example) (Miller, 1987). In this sense, we should also expand the term “New left” (as I have above) from a narrow reference to white student militants to apply to other groups breaking with the “labor metaphysic” in which workers (often implicitly male, white, and unionized) were the main or only agents of meaningful social change. It is from this angle, if any, that the new movements were often critical of the “old left” and the unfulfilled egalitarian promises and differential impacts of New Deal, which they attempted to supplement them through new analyses. Such analyses often accused the New Deal order (in many ways rightly) of being a “corporatist” collusion between state and business which promoted mass conformity and social exclusion, and opposed it in the name of individual autonomy and face-to-face community. Admittedly, by doing so, the demands of many of these groups would indeed, in the long run, inadvertently clear space for neoliberalism. The point, nonetheless, is that the political economy embodied in the New Deal—with its relative checks on capitalism in favor of social concerns—constituted the ground on which the collective demands of these movements became intelligible, powerful, and were experienced as eminently realistic—hence the dreams of psychedelic utopianism.

Thus, in sum, it was the particular post-war political economy that set the conditions of possibility for a generalized, collective upheaval amidst which psychedelics could seem revolutionary. Therefore, that hope came not only from an impersonal or accidental moment of “affluence” but also the result of class struggle within the US and abroad during the first half of the twentieth century. That struggle is what made governance and demands based on “the social” possible and greatly compressed inequality, increased the possibilities for solidarity, and fed the optimism that the world could be improved for the majority of the population. In this context, at their best, even the psychedelic counterculture's more individualist strains could contribute to a strong common sense that “the personal is political.” While elements of the *counter*culture might have taken this too one-sidedly to mean that just by changing the personal you would change the political, the simple starting point of understanding the self as socialized in harmful, oppressive and undesirable norms that are clearly identified with the operation and dynamics of capitalism is precisely what is largely missing in today's mainstream psychedelic *culture*. In all, the “democratic surge” (Crozier et al., 1973) of the 60s and early 70s built on the previous egalitarian gains of organized labor which fostered an empowering sense of security that seemed, mistakenly, irreversible. For while the “new politics” of expanded social enfranchisement would somewhat succeed in the form of what has come to be known as “identity politics” (whose achievements should not be depreciated), the material rug—and drugs—would be pulled from under them, leaving only the floating promises of individual freedom.

## Psychedelic backlash in political context

As historian Cowie (2010, 2016) has suggested, the 1970s are a sort of inversion of the 1930s—also marked by economic turmoil, they brought about the end of the New Deal coalition and the historical exception that was its period of class compromise. On the economic front, the petroleum crisis of 1973 and high government spending on the Vietnam war and social programs significantly contributed to a new situation of “stagflation” for which the Keynesian playbook seemed increasingly inadequate. Coupled with renewed international competition and progressive trade liberalization after the war, this greatly slowed down the incredible rates of national growth of the previous decade, making big business reconsider their already unhappy deal with labor and go on the offensive. Thus, the 70s witness a concerted mobilization to fight and circumvent this compromise through lobbying, union busting, pro-corporate think tanks, and by shifting production to the national or global south, where unions were weak or non-existent and labor was cheaper (a phenomenon eventually termed “de-industrialization,” and later “globalization”) (Cowie and Heatchott, 2013; Gerstle, 2022). To complete this attack on the post-war consensus, Nixon purposefully shifted the politics of the Republican party to cultural grounds, stoking and playing the rifts forming in the old Democratic coalition since its opening to the “new politics” of the sixties—that of student movements, counterculture, feminism, anti-war positions, and perhaps most importantly, racial inclusion. This last issue in particular had greatly antagonized southern democrats whose crucial commitment to the coalition rested on the defense of racial privilege as well as many white workers who were often most affected by new policies of affirmative action (Cowie, 2010). In sum, the 70s saw a situation in which attempts to expand democratic participation met with a diminishing economic pie, making it possible to break the New Deal order with a new combination of free market policies and white racial conservatism.

Considering this context allows us to view the late psychedelic controversy as more than a purely cultural matter and connected instead to the project of capitalist restoration. Even if we accept that many advocates and critics understood psychedelic use as rather apolitical and that non-political factors (such as new regulatory constraints and changing scientific standards) played a significant role in their progressive demise (Oram, 2014), there can be little doubt that at the turn of the decade, psychedelics found themselves at the heart of the struggle about the future of American Society, and were thus thoroughly politicized. One crucial element to this was precisely the association of the countercultural and psychedelic challenge to the work ethic with, on the one hand, lazy, privileged, and young troublemaking elites and, on the other, to undeserving populations (implicitly black) whose improving condition was coming through government aid instead of individual effort—all at the expense of those who had worked hard to achieve such conditions (implicitly white). Hence, in an economic climate of diminishing expectations in which the fault for stagflation was increasingly blamed on the “collectivist” policies of government welfare spending and the wage demands of organized labor (two staples of the New Deal), individualism was mobilized in the name of a conservative and pro-corporate vision of the body politic in contrast to which the psychedelic counterculture appeared as the prime example of cultural and moral degeneracy. Significantly, Ronald Reagan, who would eventually put the United States on the free market path in the 1980s, begun his rise to power as governor of California in 1966 on a platform opposing student movements, racial enfranchisement and drug use, describing a hippie as someone who “dresses like Tarzan, has hair like Jane, and smells like Cheetah” (derogatorily associating them to primitivity, femininity, and animality) (Hartogsohn, 2020, p. 179).

This platform was not entirely unlike the “law and order” track that Nixon would win his presidential election on at the boiling point of 1968, calling on the “silent majority” to oppose the new vocal minorities. Thus, his designation of drugs as “public enemy number one” was part of a broader assault on the growing social, egalitarian, and political potential embodied in those movements to which psychedelics were—uneasily, and often by their critics rather than their members—associated. As the now infamous (alleged) admission of his domestic policy advisor John Ehrlichman goes, the Nixon administration purposefully weaponized drugs to undermine the anti-war hippie left and black people (Baum, 2016). To be sure, psychedelics were *made* political by both state authorities and psychedelic activists as they confronted each other. In this confrontation, the recently unlawful status of psychedelics was certainly used as an attack on the latter. Although this move, paradoxically, might have radicalized more “neutral” users by branding them as criminals (Farber, 2002), the backlash was generally successful, for in the face of state persecution and even violence (as experienced in the protests surrounding the democratic convention in Chicago in 1968 and the anti-war demonstrations at Kent State in 1970, where four students were killed by police) it only made sense that many would drop political activism and “retreat” further into cultural rebellion (a result which also served to simultaneously further diminish political threats and increase an easily manipulable cultural polarization). “The political system,” after all, write Peter Braunstein and Michael William Doyle with regards to the decline of countercultural utopianism, “was real and hostile”—something to stay away from rather than engage with (Braunstein and Doyle, 2002).

In all, it was psychedelics' real and perceived associations to collective politics which eventually saw them demonized and sidelined in favor of the specific image of individualism shared by conservatives and—as we will see—neoliberal thinkers. Seen from this perspective, Nixon's War on Drugs was less the attempt to “blunt the counterculture by attacking its chemical infrastructure” (Pollan, 2018, p. 58) than the larger goal of breaking the New Deal infrastructure that made the collective spirit of the counterculture possible to begin with. It is perhaps the loss of that infrastructure today that skews our perception of the psychedelic counterculture by giving the sensation that what survived this loss was all it was ever meant—rather than allowed—to become. While in some ways the neoliberal order proved accommodating to certain cultural developments demanded by progressives (such as a panoply of gender and racial non-discrimination laws and a certain global cosmopolitanism), it nonetheless began as and has on the whole resulted to be a conservative reversion of the popular economic gains expressed in the post-war period that has exploded inequality and frozen social mobility (Harvey, 2005; Gilbert, 2013; Fraser, 2017).

## Capitalist realism, or neoliberalism for and against counterculture

The antagonism between neoliberalism and the counterculture is patently clear in the latter's attempt to drop out of capitalism's moral investment in work and consumption. By contrast, neoliberal thinkers sought to reinforce this investment by “economizing” ever more domains of social life, that is, by extending the rationality of the market to previously non-economic spheres and activities. Subordinating everything to the primary “project of economic growth, competitive positioning, and capital enhancement” (Brown, 2015, p. 26), neoliberal thinkers postulated that not only is a firm's main goal to grow and maximize profit, but that it is the state's goal to secure the functioning of the free market in order to foster economic growth, and, crucially, that individuals must also act rationally by seeking to maximize their own value as “human capital”—interpreting, aligning and enhancing their personal qualities and capacities in order to improve their overall competitive advantage. This meant, as Michel Foucault presciently noted in his early lectures on neoliberalism (2004), that subjects would have to behave like “entrepreneurs of the self.” The problem, which these thinkers understood, was that people did not regularly behave in such permanently self-interested ways, showing instead a propensity toward collectivism that neoliberals saw not only as mob-like and irrational but as inherently authoritarian, oppressive, and damaging to personal responsibility (an aversion to the collective which often conflated the welfare policies of the New Deal, Nazi National-Socialism, Soviet Communism, and the agendas of decolonized states) (Foucault, 2008; Gilbert, 2014; Whyte, 2019).

Their answer to this problem was that the environment had to be shaped in such a way to incentivize (read: compel) such competitive behavior. As Margaret Thatcher, who kickstarted neoliberal policies in the UK, famously put it—“economics are the method, the object is to change the soul.” This is precisely what their “policy pillars” of privatization of public goods and services, corporate and financial de-regulation, lowered (marginal) taxation, and cuts in public spending sought to achieve (Klein, 2015). As these policies enclosed the public sphere and created a sense of artificial scarcity (an ongoing, structural feature of capitalism), they successfully created the need to “procure individually what was once provisioned in common” (Brown, 2015, p. 42). This served the dual purpose of ebbing away at the conditions sustaining collective solidarity and getting people to compete, and thus work harder, instead. This had the added benefit (for capitalism, and in the eyes of neoliberals) of substituting the realm of politics—defined by agonistic battles—for that of consumption—where everyone could “vote” and express themselves through what they bought (now including goods and services to cover for their new needs), without interference from others. As Olsen (2019) has demonstrated, neoliberalism turned us into “sovereign consumers”—ostensibly free only in that realm that the counterculture had so strongly rejected.

The corollary of all this can be summed in another iconic phrase of Thatcher's, usually paraphrased as “there is no such thing as society […] only individual men and women […].”^1^ As she, and across the Atlantic, Reagan, clamped down on workers (famously, on miners and air-traffic controllers, respectively) and put their economies on a solid free market track, the work ethic, consumption and self-interested individual “freedom” were brought back with a vengeance. Thus, as inequality soared the material conditions enabling the anti-work and collective dimensions of the counterculture and other radical movements were eliminated. As Fisher noted (2018, p. 1146), neoliberalism promised “freedom *through* work,” rather than, as the counterculture had hoped, *from* it. Moreover, this elimination of the social undid the link between the personal and the political. Formally included in and considered equal by the “free market,” success or failure was increasingly understood as a matter of personal responsibility rather than of structural causes, material conditions or ideological manipulation. In all, reduced, by design, to isolated and “responsibilized” worker-consumers, everywhere subordinated to economic calculation, we might see this as the very culmination of the “technocratic society” denounced by the counterculture as a dehumanizing force. Indeed, political theorist Wendy Brown argues that in its subordination of properly political choices (such as those concerning social justice) to economic imperatives, “neoliberalism is the rationality through which capitalism finally swallows humanity” (2015, p. 44). Yet, there is also a sense in which the “humanistic” ethos of the counterculture entered into and helped revitalize the emerging economic terrain.

Although the shifts to a “networked” or “post-fordist” economic and social organization are certainly the result of broad and diverse historical and technological changes and not some purely top-down or intentional process of reactionary “capture,” it is nonetheless interesting to note how these changes were able to respond to the countercultural challenge and neatly integrate it to the operations of capitalism. As Boltanski and Chiapello showed in their classic *The New Spirit of Capitalism* (Boltanski and Chiapello, 2005), members of the managerial class took the “artistic critique” of capitalism (that opposing the regimentation, alienation and hierarchy of the world of work) seriously and developed new techniques, organizational structures, and ideological justifications in response. In place of the rigid “fordist” models of big firms and hierarchical control came the more “post-fordist” ones of subcontracting, short term work, and “horizontal” networked logistics where employees could enjoy more autonomy over their work—allowed to be both more *flexible* and more creative as they continuously shifted between new projects and connected with different people. Along with the increasing share of the service and knowledge sectors in the emerging “new economy,” this networked mode of production turned personal qualities such as “openness,” emotional intelligence, and intrinsic motivation and initiative into new assets. Formerly confined to the warm, private realm of the family or to authentic self-expression and excluded from the cold, mechanical world of work, such “human resources” now became essential to secure and ease the smooth flow of economic circuits. With them, a new image of work as a site of meaning, collaboration, and personal fulfillment—rather than something done simply for a living wage—began to take over (Jaffe, 2021).

Nowhere was this re-branding of capitalism as cool, collaborative, free, and aspirational (and its blindness to social realities) greater than in the emerging tech industry of Silicon Valley which, often abetted by old and prominent members of the 60s counterculture, was largely responsible for the additional achievement of giving technology a human face. Although Steve Jobs (who thought highly of his LSD experiences) might immediately come to mind, communications scholar Turner (2006) has traced how Stewart Brand—former fellow traveler of Ken Kesey's Merry Pranksters and author of “back-to-the-land” manual *The Whole Earth Catalog*—was central to the reconfigured perception of technology as an instrument of human liberation rather than, as it had been, of the impersonal and oppressive machinery of capitalist exploitation.^2^ Becoming the embodiment of the ideal networker in the process, Brand's multiple activities and connections were a highly influential source of the internet utopianism of the 1990s which believed that, not unlike the free market, “personal” computers and information technologies would realize the promise of a global village living in peaceful, egalitarian, and free coexistence by seamlessly connecting all individuals to each other as they finally became disembodied minds capable of unfettered agency, in the immaterial realm of cyberspace. Culminating in this apex of the counterculture-neoliberalism partnership, it was this decade that marked the final entrenchment of capitalist realism.

With the re-invigorated globalization of capitalism after the fall of the Soviet bloc, the assimilation of the global south through the economic policies of the IMF, and the acceptance of neoliberal common sense by left wing parties across the global north, it was declared that we had reached “the end of history” (Fukuyama, 1992)— the victory of capitalism and liberal democracy proved final. What is more, we had all become “middle class”– capitalism had delivered and “social critique” (concerned with equality and justice) was no longer necessary. In truth, however, as Boltanksi and Chiapello argued, as the artistic critique was incorporated and social critique was dropped, (individual) autonomy was won at the expense of (collective) security—a flimsy trade-off considering the market discipline operative in the neoliberal environment. Marked by increased inequality, the externalization of care onto individuals (mostly women, and more often of color), families, and communities (Arruza et al., 2019; Dowling, 2021), an increase in the incarceration of black bodies (at least, in the US, and largely as a result of the continuing Drug War) (Alexander, 2010), and the worsening of our climate predicament through increased extraction and consumption (despite knowledge of their consequences) (Klein, 2015; Hickel, 2021), it seems like the novel critiques of gender, race, ecology, and so forth emerging in the 60s had failed to change reality even if they had in fact changed culture, and that the world remained as mad as ever. In another reversal, however, that madness was turned “straight” again by the “decade of the brain”—and so were psychedelics.

## Situating the psychedelic renaissance

The point of view I have developed so far allows us to see the coincidence of the psychedelic renaissance, the definitive rise of neuroscience, and neoliberal hegemony as more than accidentally connected (even if not inherently so)—for they all have in common a focus on the individual at the expense of social context that serves to obscure the latter's effects as well as its contingency.^3^ While a full exposition of their connections is impossible here (see Cohen, 2016), we can briefly note how, concretely, the three converge on what Mark Fisher termed “the privatization of stress,” by which he referred to the ways in which mental health issues were emptied of their social meaning and disconnected from their social causes (2018). For Fisher, the main agent of this privatization was the medical model of mental illness (2009, p. 21), which conceived it as the result of “chemical imbalances” in the individual brain which could be directly targeted and compensated through psychiatric drugs. Interestingly enough, psychiatry only fully embraced this model since the publication of the DSM III in 1980 as a response to a growing sense of crisis due, not least, to the popularity of “anti-psychiatric” critiques that saw it as an institution for the social control of deviance, its drugs as mere means to render patients docile, and madness as in fact a sane response to an oppressive world (evincing, remember, the synergies between psychoanalysis and social critique at the time) (Whitaker, 2010; on the turn to biopsychiatry, see also Harrington, 2019). Again, psychedelic's contribution to this challenge to psychiatry has been documented by Hartogsohn (2020, p. 231–241), who shows how the valorization of psychedelic altered states as positive and healthy deviations from the narrow strictures of “normal” consciousness drew from and informed a similar revalorization of madness (to which psychedelic consciousness has long been compared). Pertinently, the term “acid communism” originated in a documentary about Scottish psychiatrist Ronald D. Laing, a prominent if controversial anti-psychiatric figure who also experimented with LSD therapy and argued that madness may not all be breakdown, but also “breakthrough” to better ways of being (Laing, 1967).

This is to say that, just like neoliberalism and in parallel to its historical ascendance, the medical individualization of personal distress also occurred as a reaction to a period in which, as Staub (2011) has put it, “the diagnosis was social.” Not least, notes Staub, this reaction repeatedly expressed a “fashionable kind of slander” (p. 167) aimed at activists and hippies who stood accused of celebrating craziness, being mentally ill-adjusted themselves and of having impeded those in need of treatment from getting it. As this narrative took hold, enormous funds were channeled into new drugs and technologies that promised direct knowledge of and intervention on brain mechanisms. Thus, as the idea that it was *society* making people sick and therefore what was in need of transformation was discredited, it was finally possible to bring psychedelics back into the psychiatric fold. As Nicolas Langlitz argued ten years ago, “it was the neuroscientific disenchantment and depoliticization of hallucinogen research that rendered its revival possible” (2013, p. 45)—and this was itself a political maneuver.

Far from neutral, then, contemporary psychedelic science has always been political in its disavowal of its countercultural legacy (even as it lives off of it). This neutrality is further questioned by the epistemological tension between its methods and psychedelic experience, for in its heuristic individualism and abstraction of experience from context, neuroscience directly contradicts the continuous insistence on the importance of “set and setting” to the latter. Thus, the main task neuroscience was summoned to complete was to “contain” (Noorani, 2021) psychedelics and render them neutral with respect to a particular—neoliberal—background. By limiting psychedelics' potential to helping individuals adapt to, rather than challenge, a social world that it takes as given, neuroscience reproduces capitalist realism. In other words, while claiming itself free of the ideological add-ons of both left-wing counterculture and (now also) right-wing conspirituality (Pace and Devenot, 2021), and to be “demystifying” (Carhart-Harris et al., 2018). New Age spirituality by grounding it in the brain, the neuroscientific approach to psychedelics has in fact reproduced and naturalized the individualist assumptions of its own surroundings while hiding this very operation (for some, the very definition of ideology). Along the way, admittedly, it has altered—with some promise—the conception of mental illness from one focused on chemical imbalances to that of overly rigid neural pathways, but in this, it has merely “swapp[ed] out one biomechanical model of the diseased brain for another” (Devenot et al., 2022, p. 487). If anything, this new “connectionist” model of mental health reinforces neoliberal norms to an even further degree.

This is because, whether a matter of enhancement or treatment, the effects on the brain (and the mind) that psychedelics are praised for—“openness” (MacLean et al., 2011),^4^ “connection” (Carhart-Harris et al., 2018), “flexibility” (Sloshower et al., 2020), “creativity” (Mason et al., 2019), “flattening” of hierarchies (Carhart-Harris and Friston, 2019), etc—exactly mirror the demands that neoliberalism's networked mode of production makes of its subjects (Malabou, 2008). On the one hand, considering the new competitive pressures of the “neoliberal rat race,” it is little surprise that the revival of psychedelic research coincided with a problematization of enhancement in public discourse (Langlitz, 2013, p. 233). Even less surprising is that the best example of this lies in Silicon Valley, historical core of the counterculture and its utmost neoliberal instantiations, and finds expression in the hype around microdosing psychedelics in order to boost creativity, energy and interpersonal openness—read: productivity—at work (Kuchler, 2017). Quite literally taking the edge off of a “full blown” psychedelic experience that can prove difficult, confronting, and hard to control, this practice manages to seamlessly integrate the benefits of psychedelics into the smooth circuits of contemporary capital.

A similar logic might be said to inform, on the other hand, the use of psychedelics for treating of a variety of mental health issues for which psychedelic “connection” and “plasticity” may have transdiagnostic value (Carhart-Harris et al., 2018; Kočárová et al., 2021). This may be especially true in the case of depression, which has been deemed the characteristic pathology of our neoliberal era by Fisher (2018) and a number of other cultural commentators (van den Bergh, 2012; Rogers-Vaughn, 2014; Rosa, 2019). Often described as a radical form of disconnection (Watts et al., 2017; Hari, 2018) and loss of agency, depression stands as the diametrical opposite of the norms of networked connectivity and constant self-actualization—quite literally an expression of the exhaustion of having to perpetually (im)prove oneself (and symptomatically indistinguishable from burnout) (Ehrenberg, 2010; Bianchi et al., 2015). For philosopher Han (2015, 2017), the spread of depression and burnout reflect the ways in which today we are coerced through freedom—in other words, how, in the apparent absence of obstacles yet under pressure to conform to market logic at every step, we assume responsibility for doing more and better, or failing to do so.

The promise of psychedelic therapy is that, by inducing neuronal and psychological flexibility to undo the rigid cognitive, neuronal, and behavioral patterns of patients, it can help connect patients back to the world (Watts et al., 2017; Sloshower et al., 2020; Watts and Luoma, 2020). This is a commendable goal that can indeed help many deal with or overcome harrowing forms of distress. However, to the extent that the site of these psychedelic interventions always remains the individual and they do not question the broader social processes driving disconnection (or psychological and neural rigidity), they remain caught within responsibilizing, or “neuro-responsibilizing” (Biebricher, 2011), horizon of capitalist realism (see also Illouz, 2008). The point is not to be “against” such forms of treatment but to point to the fact of their limited horizon and to the contradiction between a discourse of connection and the material realities (an atomizing, competitive environment) and healing models (focused on the individual) of disconnection. It is also to suggest that, as “different dimensions of inequality increase” and generate “forms of mental distress […] that are becoming more and more common,” another psychiatry—one more focused on social determinants and solutions—is possible (World Health Organization, 2014; Rose, 2019; Petrement, 2023).

## Conclusion

This essay has briefly tracked the journey of psychedelics from countercultural tools of liberation from capitalism to mainstream medicines of integration into it, and historicized this transition within changes in political economy associated with and grouped under the term “neoliberalism.” Following the work of Mark Fisher, the intention behind this historical investigation has been to recover something that has been all but lost—the collective potentials embodied by the psychedelic counterculture and other social movements of the 1960s, and perhaps more importantly, the conditions that made them possible. Stemming from a widely acknowledged need to curb the destructive effects of capitalism in name of social and public needs, the New Deal order brought about a class compromise and compression of inequality that (along other factors) sustained a sense of economic security. Enabled by such “affluence” while trying to correct for its racial and gendered exclusions, new social movements expressed an optimistic confidence that further egalitarian and democratic social change was possible. In that context, psychedelics (notably LSD) seemed to promise a revolution of consciousness that could inform a more humane culture, one free of both hierarchical social divisions and of the compulsion to work and consume. It is in reaction to these material conditions and collective potentials that the neoliberal project took off.

Emerging as a conservative cultural and economic backlash promising to defend and support individual freedom and effort from collectivist entitlement, neoliberalism also managed, in the long run, to incorporate countercultural values into its economic functioning. Although the counterculture had a clear individualist streak from the beginning, the networked and competitive environment of neoliberalism has turned “psychedelic” values such as autonomy, connection, openness, creativity and flexibility into norms to comply with as much as, or even rather than, expressions of personal freedom. Ignoring matters of political economy and suggesting that success or failure to comply with these norms stems from individuals' neural and mental rigidity—which can be treated or enhanced through psychedelics—rather than from environmental pressure, the neuroscientific and medical approaches dominating the psychedelic renaissance reproduce the responsibilizing logics of neoliberalism (Gearin and Devenot, 2021). In so doing, they de-link the personal from the political and conceal the crucial insight of consciousness-raising: that the self is a product of socialization, and thus that not only does the social very much exist (contra Thatcher), but that we must aim to transform the world around us if we are to become who we desire to be. Considering that, as mentioned above, the gendered, racial, and ecological problems and divisions that radical movements in the 60s brought attention to and sought to overcome are still with us (if in mutated form) (Fraser, 2022), we would do well to adopt that insight and learn from their experience.

With respects to the psychedelic counterculture, we should certainly pick up on its rejection of work and consumption and its opening of the privileged middle classes to a broad and downwards tending social solidarity, and indeed reject its individualist politics of authenticity. First, the anti-work and anti-consumption ethos of the counterculture could help us detach our sense of personal worth from the work we (or others) do. In other words, it could stop us from pinning judgements about those who “deserve” decent lives on the kind or amount of work they do and on how they spend their income on, which would open up space for solidarity with those considered "undeserving". Second, while back then such an ethos mostly hinged on their harm to personal freedom, authenticity, and intrinsic dignity, some would argue that today it has also become an ecological imperative (Levitas, 2008; Hickel, 2021). Thus, remaining critical of the consequences of our regime of work and consumption might also help us think about the material consequences of these activities and ideological investments. Of course, neither of these shifts are easy to enact voluntaristically in a competitive context, but that is the whole point! - it is such context which impedes us from such “personal” changes. On the one hand, it has become increasingly difficult to dis-identify from work as it takes more of our time and we have come to “love” what we do (Jaffe, 2021), and such lack of time translates into more intensive forms of consumption (e.g., flying instead of taking a bus, or buying pre-packaged food instead of cooking). On the other hand, while in the 60s, some may have been able to identify downwards due to a profound sense of economic security, neoliberalism undid that possibility by instilling in the middle classes a “fear of falling” (Ehrenreich, 1989) that inspired us to identify upwards instead. Ironically, as the predominantly middle class constituency of Occupy Wall Street and other “new social movements” exemplifies, this might have started to go far enough to have to reversed such identification again by undercutting upward mobility.

Finally, this experience should remind us of the contextual constraints of individual agency—which is never absolute and disembodied but always tied to one's social position. Contrary to popular self-help ideologies still permeating psychedelia (Plesa and Petranker, 2022), no one can transcend these limits by sheer act of will or enlightened self-awareness, for they are not (merely) the product self-limiting or culturally imposed beliefs under which one can find a source of boundless freedom. Of course, this individualist ontology was not invented by the counterculture but has a much longer history associated with liberal humanism and capitalist modernity (Gilbert, 2014), and it is indeed associated to the gendered and racial hierarchies the counterculture was often blind to. As many have noted, the Cartesian separation of the rational mind [and, nowadays, brain (Ehrenberg, 2004; Vidal, 2009)] from the natural world and the physical body allowed for the scientific instrumentalization of nature and the theory of autonomous agency, and this often translated into the instrumentalization and denial of agency of gendered and racialized others through their association with the body and nature (Plumwood, 1993; Braidotti, 2013).^5^ Along with the disciplining of the body, conceived as a mere machine, according to new norms of “productive” subjectivity, these divisions have been instrumental to capitalist accumulation since the very beginning (Federici, 2004). From this longer point of view, the surprising thing is not that the psychedelic counterculture reproduced the pitfalls of this heritage and could not, in the last instance, break free from it, but that at a certain moment and under particular conditions—characterized by a relative relaxation of capitalist demands—it could—ever so briefly, in some ways, yet powerfully - point beyond it. Challenging the close connection between the scientific and technological worldview, capitalist exploitation, and social divisions, and seeking instead to place the human self within its larger cosmic and natural surroundings—this is still a project worth picking up, and to which psychedelics can still powerfully contribute (see also Falcon, 2021).

Provided we follow their wisdom and do not dissociate experience from its context, psychedelics remind us to struggle for the “collective set and setting” under which an emancipatory and ecological project traversing the mental, social, and natural can materialize (Guattari, 2000). In turn, the sensitivity of psychedelic experience on its “rhetorical conditions” (Doyle, 2011) reminds us that they will contribute to this project if and only if we summon them to do so and think more deeply about their place within it. This possibility is foreclosed by appeals to prudent neutrality which themselves seem to adhere to a belief in the essential benevolence of psychedelics, as if simply getting them mainstreamed will result in a better world (Davis, 2018). Again, this is not to be against the healing power of psychedelics. On the contrary, it is to take it seriously, for as cracks appear in the neoliberal order (Gerstle, 2022), the divergence of the therapeutic mechanisms of psychedelic therapy from individualist conceptions of mental health could once again become a turning point in our common sense ideas about the relationship between self and world, teaching us that personal experience—whether it be psychedelic, “mentally ill”, or everyday—is always political. In other words, all experience—not just that of a psychedelic type-is shaped by broader forces that are not neutral or immutable but contingent and shaped by power relations.

As several participants in clinical trials with psychedelics insisted during the International Conference on Psychedelic Research (ICPR) last year, it is time that we treat people like plants: for “if a plant were wilting we wouldn't diagnose it with “wilting-plant-syndrome”—we would change its conditions. Yet when humans are suffering under unliveable conditions, we're told something is wrong with us, and expected to keep pushing through” (Ahsan, 2022). Hopefully this essay can contribute to our thinking in such ecological way about our social conditions, and psychedelics can help us change them.

## Data availability statement

The original contributions presented in the study are included in the article/supplementary material, further inquiries can be directed to the corresponding author.

## Author contributions

The author confirms being the sole contributor of this work and has approved it for publication.

## Footnotes

^1^The real quote goes “society? There is no such thing! There are only individual men and women, and their families” (Jaffe, 2021) and as Dowling (2021) notes is followed by an often ignored recognition that there is “a living tapestry” of people on whose ability and willingness to help each other depends their quality of life. In other words, there *is* a certain conception of the social or of communal (Rose, 1996) linked to neoliberal governmentality, but it is still one based on personal responsibility rather than publicly managed and funded care, and on conceptions of collectivities as mere aggregates of individuals (Gilbert, 2014). This is an important point that this essay has not been able to delve into, and complements the expanded role of notions of “connection” not only in the realm of work, but also that of social relations—in both cases, the more disconnection prevails, the more connection is summoned. Nonetheless, the displacement, “dismantling,” or “hollowing out” of the social is still a common theme of several critical analyses of neoliberalism and our digital era (Brown, 2019; Couldry and Mejias, 2019).

^2^Thanks to the reviewer for pointing out the longer conversation about this counterculture-capitalism mix under what Richard Barbrook and Andy Cameron termed “the Californian ideology” (Barbrook and Cameron, 1996), where they trace the strange “amalgamation of opposites” (new left and new right, hip and yuppie…) of the emerging “virtual class.” In this essay, I have tried to acknowledge this “hybrid” while trying to highlight their originally distinct (“opposite”) forms.

^3^Granted, this is not (for lack of space) a very sophisticated or in-depth critique of neuroscience, and many will roll over their eyes at another “external” critique of neuro-reductionism. Certainly, questions remain about the possibilities opened by “social neuroscience” and the extent and form of the uptake of neuroscientific discourse by the public at large. Nonetheless, on the whole I believe the critique of the individualizing logic of neuroscience (and biopsychiatry) still remains as necessary as ever given its problematic history (in relation to financial interests, institutional support, and often dubious merits) and current authority. While certainly, as Rose (2019) suggests (Rose and Abi-Rached, 2013), neuroscience might still help ground claims regarding the effects of social environments, my overarching concerns are that whether in requiring all such arguments to “pass through the brain” we are not in fact losing the ability to listen to those in distress (as his own reference to Foucault would seem to prioritize) unless they can access, translate, and voice their concerns in the language of neuroscience and that we may be reproducing problematic hierarchies of knowledge in the process (e.g., subordinating social modes of knowledge). On another note, as Langlitz (2013) traces, neuro-reductionist discourse has a precedent precisely in Timothy Leary. Nonetheless, Leary's horizon and project, unlike much of today's neuroscience, was very much one of social transformation—especially after the criminalization of psychedelics that saw him persecuted and incarcerated by the state and his subsequent contact with the Weathermen and the Black Panthers, through which this dimension became more explicit for him.

^4^Interestingly, in such “openness” is often related—like in the study about the ecological and political dimensions of psychedelic experience (Lyons and Carhart-Harris, 2018)—to liberal as opposed to conservative political views. Although of course coming from and belonging to completely different academic disciplines and intentions, it is arguable that this sort of framing obscures the existence of a position “left of liberal” and any investigation of the relation of openness or psychedelic experience to it.

^5^From this point of view, it is interesting that the period of class compromise embodied in the New Deal is sometimes referred to as that of “embedded liberalism” (Cowie, 2010)—a qualifier suggesting the disembeddedness of liberalism as a whole and also somewhat obscuring the socialist forces that pulled it down to earth.
